# Superspreading quantified from bursty epidemic trajectories

**DOI:** 10.1038/s41598-021-03126-w

**Published:** 2021-12-16

**Authors:** Julius B. Kirkegaard, Kim Sneppen

**Affiliations:** Niels Bohr Institute, University of Copenhagen, 2100 Copenhagen, Denmark

**Keywords:** Computational biophysics, Applied mathematics, Statistics, Diseases, Population dynamics

## Abstract

The quantification of spreading heterogeneity in the COVID-19 epidemic is crucial as it affects the choice of efficient mitigating strategies irrespective of whether its origin is biological or social. We present a method to deduce temporal and individual variations in the basic reproduction number directly from epidemic trajectories at a community level. Using epidemic data from the 98 districts in Denmark we estimate an overdispersion factor *k* for COVID-19 to be about 0.11 (95% confidence interval 0.08–0.18), implying that 10 % of the infected cause between 70 % and 87 % of all infections.

## Introduction

In controlling epidemics, a deep understanding of the dynamics that underlie the spread of a disease is critical for choosing which interventions are most efficient to mitigate its continued spread. Epidemiological models of disease spreading^1,2^ depend on parameters that capture effects both of the pathogen–host biology^3^ and the behaviour of the population in which the disease propagates^4^. Population-level data allow the estimation of the *average basic reproduction number* *R*, denoting the average number of people an infectious individual will transmit the disease to. Hidden in the average value of *R* are both temporal variations and variations between infectious individuals^5^. Variations in time stem both from the fact that social behaviour can change during an epidemic due to e.g. interventions being put in place, and because as the epidemic progresses the susceptible fraction of the population decreases. Variations from person to person can results both from biological differences or social behaviour.

**Figure 1:**
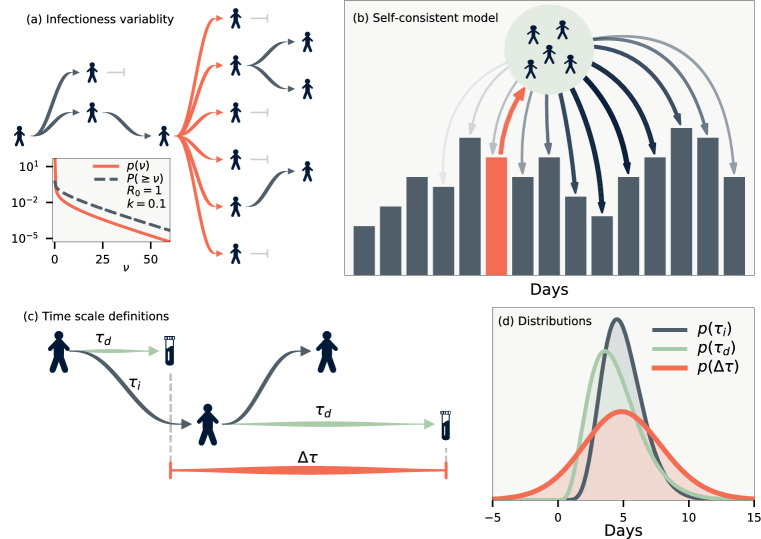
Model definitions. (**a**) Illustration of a heterogeneous infection pattern (superspreading). Inset shows the probability density function and (one minus) the cumulative probability for the gamma distribution $\Gamma (R=1.0, \, k=0.1)$. (**b**) Likelihood model. The infected individuals whose infection was reported on some day (orange) will themselves infect a number of people. These are in turn detected on other days according to the distribution $p(\Delta \tau )$. (**c**) Time scale definitions. $\tau _d$ denotes the duration from being infected to being reported, and $\tau _i$ the duration between infections (generation time). Finally, $\Delta \tau$ is the difference between the reported times of infector-infectee pairs. (**d**) The maximum likelihood of the distributions we employ for $\tau _d$ and $\tau _i$, and the distribution thus implied for $\Delta \tau$. $p(\Delta \tau )$ has support below zero as it is possible that the infector’s infection is reported after the infectee.

A popular tale for some diseases is the 20/80-rule stating that 20 % of infectious individuals are responsible for 80 % of all infections. This was for example seen in recent epidemics such as the 2003 Asia outbreak of SARS^5^ and the 2014 Africa outbreak of Ebola^6^. Numerous of studies of COVID-19 suggest even more extreme statistics for this disease^7–11^. These effects have collectively become known as *superspreading*, and while it is simple to define theoretically, measuring them typically requires data at the level of individuals. Viral genome sequences can be used to inform the analysis^12,13^, and when contact tracing data is available^14–16^ the analysis may be performed directly. More indirectly, the number of imported versus local cases has also been shown to inform the dispersion^17^. Focusing exclusively on the early evolution of the epidemic, recent work^18^ has shown that the variation in infection rate between regions can be used to estimate the dispersion.

In this report, we derive a Bayesian model for local epidemic outbursts to address the inverse problem of estimating temporal variations and individual infection heterogeneity from aggregate data. In other words, we demonstrate how to estimate this heterogeneity using data that only contain the total counts of the number of infected (and tested) per day. Our method relies on the fact that the epidemic trajectories of case numbers are bursty on a regional level, reflecting a mixture of simple Poisson randomness, varying testing frequencies, and individual infection heterogeneity. We model these fluctuations and sample for the statistics of the duration between reported cases as illustrated in Fig. 1). Using regional data allows us to bypass the averaging on the larger scales and permits the estimation of the underlying heterogeneity. Our method simultaneously samples across many regions, and thus naturally separates local outbursts from the large scale variation in average reproduction number.

**Figure 2:**
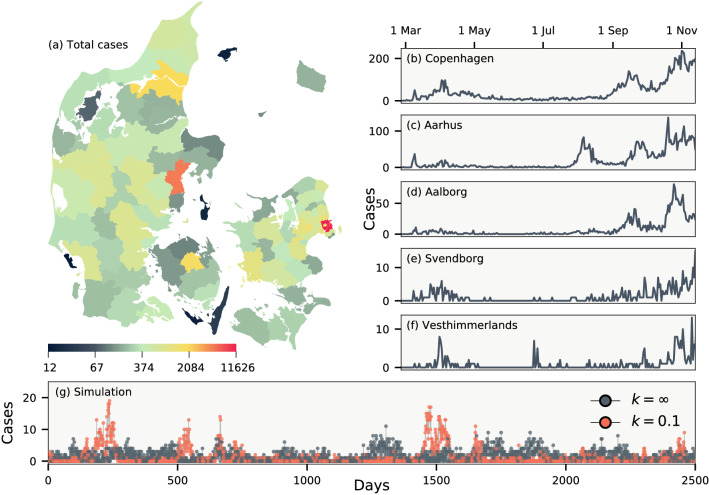
Daily cases of COVID-19 in Denmark between 26 February and 17 November 2020, during which only PCR tests were employed. (**a**) The total number of cases in each of Denmark’s 98 municipalities. (**b**–**f**) Daily number of cases in five municipalities. (**g**) Simulations of an epidemic with dispersion parameter $k = \infty$ and $k = 0.1$, respectively. Both simulations use $R = 0.9$ and a crossing parameter chosen such that on average an infectious person enters every fifth day. Map created from DAGI [“Danmarks Administrative Geografiske Inddeling”] data (2020) supplied by the Danish Agency for Data Supply and Efficiency.

We apply our approach on data for Denmark, which has a number of features that permit the analysis: Denmark, with a population of 5.8 million, makes available daily data for all of its 98 municipalities, which all coordinate their testing identically. As shown in Fig. 2(a–f), the number of cases in these municipalties vary significantly. In the capital region of Copenhagen, daily cases number in the hundreds, whereas in more rural Vesterhimmerlands the daily rate is less than ten. Finally, the population of Denmark is fairly uniform and thus slow, temporal variations in *R* can be assumed to affect all regions.

## Model

Following the seminal paper of Lloyd-Smith et al.^5^, we assign each person an infectivity $\nu$ sampled from a gamma distribution $\Gamma (R(t), k)$ with mean *R*(*t*) and dispersion parameter *k*. Small *k* correspond to a disease driven mainly by superspreading as illustrated in Fig. 1(a). The mean basic reproduction number is taken to be time-dependent to include changes due to policy, behavior and immunity. Accounting for subsequent independent stochastic infections, the offspring distribution is negative binomial $\text {NB}(R(t), k)$^5^. Our objective is to estimate *R*(*t*) and *k*. These parameters can be deduced directly if contact tracing data is available. Using only aggregate data, however, we need to instead build a probabilistic augmentation of the missing contact information.

In aggregate data, the duration between the infections of an infector-infectee pair being reported $\Delta \tau$ is stochastic. This distribution can be calculated if the distribution of infection-to-infection $p(\tau _i)$ (generation time) and infection-to-reporting $p(\tau _d)$ are known. As illustrated in Fig. 1(c), the time between reporting obey the random variable relation $\Delta \tau = - \tau _d + \tau _i + \tau _d$, where the first $\tau _d$ refers to the random time of reporting for the infector and the latter $\tau _d$ the time of reporting for the infectee. These do not affect the mean value of $\Delta \tau$ but do increase its variance. The resulting distribution is shown in Fig. 1(d) using estimates from the literature of $p(\tau _i) \sim \Gamma (5.0 \pm 0.75, \, 10 \pm 1.5)$ and $p(\tau _d) \sim \Gamma (4.5 \pm 0.75, \, 5.0 \pm 1.0)$^19,20^. With these distributions in place it is straightforward to simulate an epidemic if *R*(*t*) and *k* are known. Fig. 2(g) shows two such examples for $k = \infty$ and $k = 0.1$. For $k = 0.1$ there will be superspreading, but because of the distribution of $\Delta \tau$ these will be distributed over a number of days rendering visual distinction difficult and thus makes statistical analysis crucial for its discovery.

**Figure 3:**
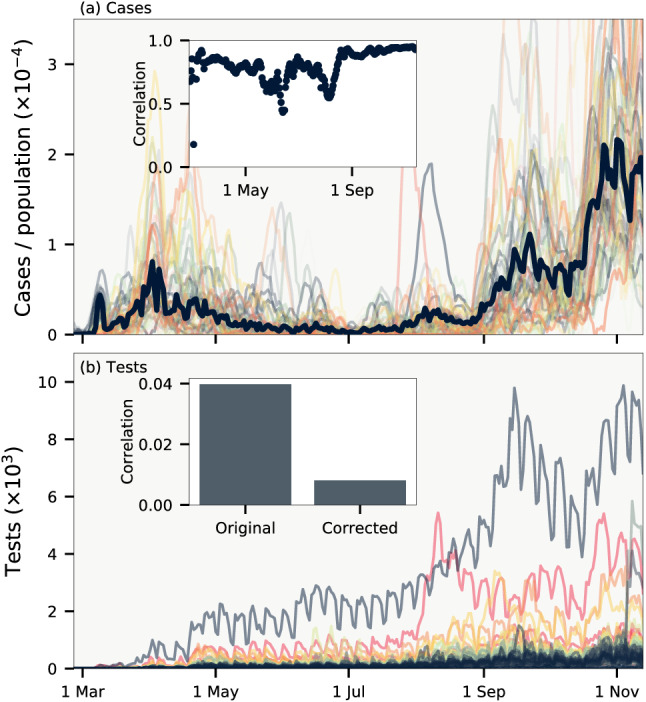
Data correlations. (**a**) Cases per population of each municipality smoothed over one week. Thick line shows cases for all of Denmark. Inset shows the cross correlation between municipalities as a function of time. (**b**) Daily test frequency in each municipality. Inset shows the correlation of deviations of daily cases from a weekly running mean both with and without linear correction for the number of tests.

To tackle the inverse problem of the simulation we define a self-consistent model of the data. For simplicity let us first assume that all infectious individuals are found and postpone the discussion of under-reporting. Figure 1(b) illustrates our approach: we define the likelihood of the data by calculating the probability of the observed time-series for each municipality. In practice, this can only be calculated in a reasonable amount of time because of a few key features of the negative-binomial distribution. These are derived in the methods section, but can be summarised as follows: If the offspring distribution from a single individual is the negative binomial $\text {NB}(R, k)$, then the offspring distribution from *M* people, where each individual is found on one specific day with probability *p* is exactly $\text {NB}(p\,MR, M k)$. The total likelihood of a single day is then found by convolving these distributions using $p(\Delta \tau )$ for the daily probability of reporting. The precise formulae are presented in the SI.

To complete our model, we need to adjust for correlations that are present in the data as shown in Fig. 3. Naturally, a municipality with a large population will have a larger number of cases per day than a municipality with a small population. This is because there will be more imported cases in large regions (there may also be variations in *R* between cities and rural areas^21^, but this is a second-order effect that we ignore). As most imported cases will come from other municipalities, we ignore effects of international travel. In fact, daily cases per population of the municipalities will be strongly correlated as a function of time as demonstrated in Fig. 3(a), reflecting the fact that Denmark is a small country with overall homogeneous development of the disease. To account for coupling between communities we introduce a crossing parameter *c* that corrects for the fraction of infections that occur across municipality borders. For the number of infectious individuals in municipality *m* we thus use $N_m^\text {corrected} = (1 - c) N_m + c f_m T$, where $N_m$ is the uncorrected number of infectious individuals in municipality *m*, $f_m$ is the population fraction of municipality *m*, and *T* is the total number of infectious individuals across all municipalities. With this simple formula it is ensured that municipalities will, on average, have a number of infections that is proportional to the population of the municipality.

Figure 3(b) shows that the testing frequency in each municipality is highly irregular, with e.g. fewer tests being done on weekends. Our method to detect variations in reproduction number depends on the deviations in cases in each municipality to be uncorrelated. The inset of Fig. 3(b) shows that there is a small correlation present. This is natural, since the number of tests is correlated across municipalities. We correct for this effect by scaling with the number of tests. This is incorporated into our model by re-scaling the distribution of reporting $p(\tau _d)$ in proportion to the daily number of tests (see SI for details).

**Figure 4:**
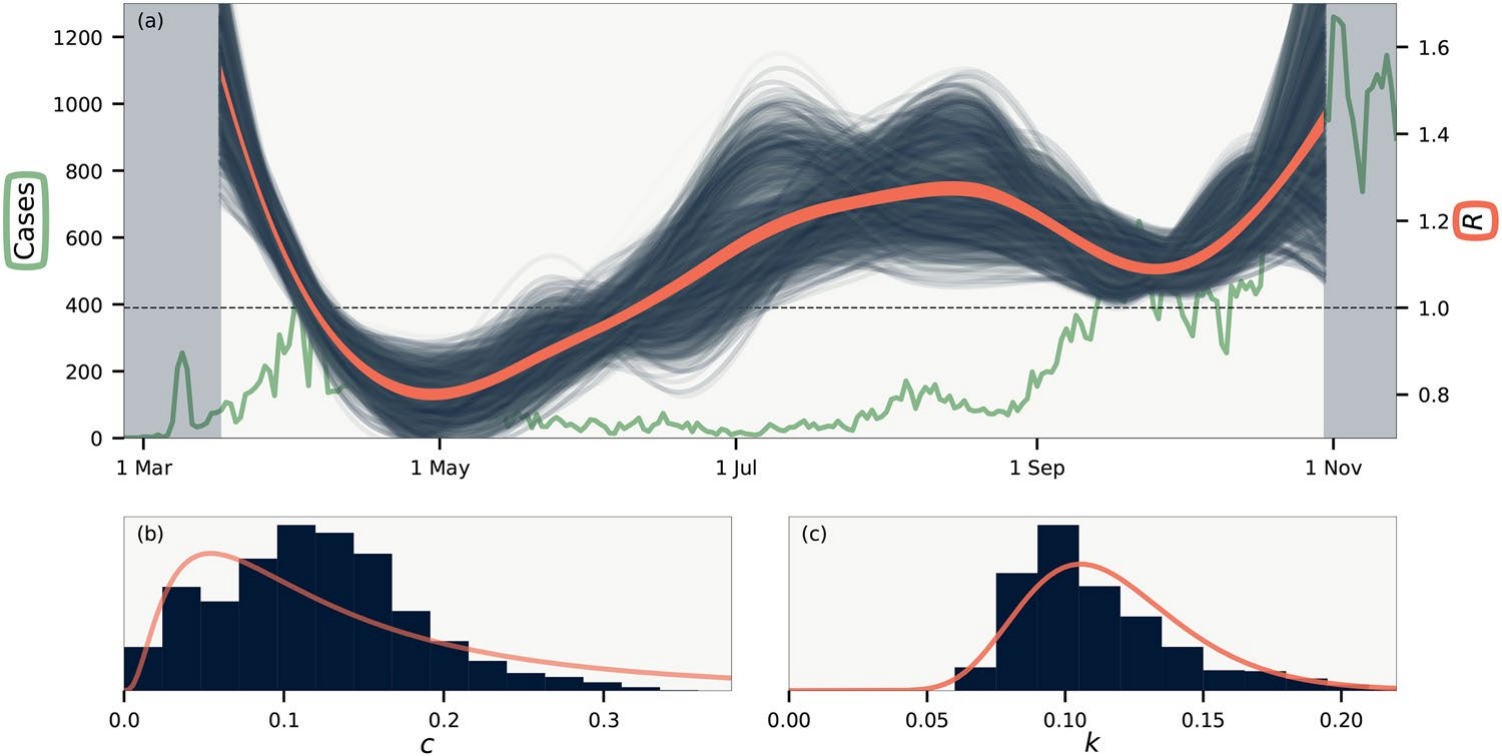
Results. (**a**) Temporal variations of the basic reproduction number *R*(*t*). Background line shows total number of daily cases. Blue lines are realisations from the MCMC sampling, while orange line indicates average of all samples. Shaded background shows sections of the data that are not included in the likelihood. (**b**) Histogram of the crossing parameter *c*. (**c**) Histogram of the dispersion parameter *k*. Curves in (**b**–**c**) are log-normal distributions with matching mean and variance.

Finally, we employ Hamiltonian Monte Carlo^22^ to sample for *R*(*t*), *k* and *c* from the total likelihood function of all regions, aimed to reproduce the case counts at each day, given case counts on previous days. In particular, we run the NUTS algorithm^23^ with gradients of the log likelihood calculated by automatic differentiation^24^ on GPUs that allow for fast calculations of convolutions that make up our likelihood function (see methods). We restrict temporal variations of *R*(*t*) to be slow on the scale of weeks by parameterising the function using cubic Hermite splines. The Hamiltonian Monte Carlo chain is then run multiple times for sampled $p(\tau _i)$ and $p(\tau _d)$.

## Results

Our results are shown in Fig. 4. The sampling reveals an *R*(*t*) [Fig. 3(a)] that slightly deviates from estimates obtained by single approximations using e.g. the SIR model^1,25^. This is because we calculate an *R*(*t*) that best explain the statistics of each municipality and not the sum of these. Further, we have a large uncertainty on our estimates because our *R*(*t*) models the reproduction number under uncertain values of $p(\tau _i)$ and $p(\tau _d)$ (see Ref.^26^ for details on precise estimation of *R*(*t*) alone). In other words, we calculate the true value of *R*(*t*) as defined by the average offspring count, and not as the value of *R*(*t*) that best makes a single model fit the evolving infection statistics^27^.

Figure 3(b) shows that we cannot constrain *c* more than to say that by far most infections happen within municipality borders, as is expected. In contrast, the degree of superspreading as defined by the value of *k* is fairly constrained as shown in Fig. 4(c). We find *k* in the range 0.08 – 0.18 (95% confidence interval), with mean $k = 0.11$, which compares well to estimated confidence intervals obtained from other methods^12,14,15,17,18^ but smaller than $k \sim 0.4$ reported by Ref.^16^. For $R = 1.4$, for instance, our value range corresponds to an epidemic in which $10 \, \%$ of the infected individuals are responsible for $70 \, \%$ – $87 \%$ of all cases. In this case, the majority of infectious individuals will not infect anyone, in broad agreement with the fact that there are remarkably few transmissions within households^28,29^. We note that the precise range of such statistics depends on the choice of probability distribution for infectiousness, for which we used the gamma distribution as has become standard^5^. If, for instance, the distribution instead were fat-tailed^4,9^, then the quantification of the dispersion statistics would differ. This could be remedied by introducing an exponential cutoff, but then this extra parameter would also need to be sampled for. The value of the crossing parameter *c* only weakly affects *k*, which is instead affecting mainly by the mean value of $\tau _i$ and the widths of the distributions of $\tau _i$ and $\tau _d$. In particular, in our model we assume that infectious individuals spread the disease over time. If, in contrast, the spread from individuals is driven mainly single events then our distribution for $p(\Delta \tau )$ is too wide. To study the effect of this, we ran our model with both $p(\tau _i)$ and one of the two $p(\tau _d)$ that make up $p(\Delta \tau )$ constrained to a single day. This leads to a *k* that is about $40 \, \%$ larger than the one estimated.

We have until now assumed that all infectious individuals were included in the data. This is of course not true. Focusing on estimating *k* we here consider the case where only a (time-independent) fraction $f<1$ of all infectious are found. This leads our method to overestimate *k*. Most simply, if the incidence at each day is a factor 1/*f* larger than the measured data, fluctuations are amplified by 1/*f* and the true dispersion parameter *k* will be our measured *k* multiplied by *f*. Thus a value of $k=0.1$ from Fig. 4c and an $f\sim 1/3$ would correspond to a true $k\sim 0.03$. It is however more realistic to assume that each detected case is independently found with probability *f*. Using simulated data where a fraction $f \sim {1}/{3}$ of cases are independently detected we find that a measured *k* of 0.1 correspond to a true underlying *k* that is between 0.05 and 0.085, depending on the simulation (see SI). If, on the other hand, there is large correlations between the reporting present in the data, our method may underestimate *k*. This is harder to gauge precisely as it depends on the correlations.

These systematic uncertainties should be considered for our estimated value of *k*. The existence of large spreading events makes our model underestimate *k*, whereas uncorrelated under-reporting leads to overestimation. The effects will tend to affect the value of *k* in opposite directions, but taken to the extreme could bring *k* to 0.04 – 0.28. We have furthermore tested our method on random subsets of all municipalities, and found that this did not have any significant impact on our estimates. Restricting to considered time interval to smaller subsections also did not affect the estimated value of *k* significantly.

Traditionally one characterises an epidemic with only one number, $R_0$, and even so there are remarkably few direct measurements of this average for known diseases. Here we ventured beyond such average measurements and proposed a new community level method to extract also variations in infectivity without having access to person sensitive data and contact tracing. Using our method we quantified the COVID-19 epidemic as one of the most extreme superspreader dominated diseases ever recorded^5^. It has previously been demonstrated that such level of heterogeneity should make COVID-19 comparatively easy to mitigate with societal restrictions^10,11^.

## Methods

### Negative binomial formulas

The offspring distribution from an individual with infectivity $\nu$ is Poissonian:

1 $$\begin{aligned} p(n) = \text {Pois}(n ; \, \nu ) = \frac{\nu ^n \, e^{-\nu }}{n!}. \end{aligned}$$

When infectivty $\nu$ is distributed according to a Gamma distribution

2 $$\begin{aligned} p(\nu ) = \Gamma (\nu ; \, R, k) = \frac{\nu ^{k-1}}{\Gamma (k)} \left( \frac{k}{R} \right) ^k e^{-\frac{k \nu }{R}}, \end{aligned}$$

the total offspring distribution becomes negative binomial

3 $$\begin{aligned} p(n) = \text {NB}(n ; \, R, k) = \frac{\Gamma (n + k)}{n! \, \Gamma (k)} \frac{k^k R^n}{(k + R)^{n+k}}. \end{aligned}$$

The negative binomial has probability generating function

4 $$\begin{aligned} G_n(s; R, k) = \left[ 1 + \frac{R}{k} (1 - s) \right] ^{-k}. \end{aligned}$$

The number of infections from *M* infectious people will then have generating function

5 $$\begin{aligned} G_n^M(s) = \left[ 1 + \frac{R}{k} (1 - s) \right] ^{-M k }= \left[ 1 + \frac{MR}{M k} (1 - s) \right] ^{-M k} = G_n(s ; M R, M k). \end{aligned}$$

If a person is only reported with probability *p*, corresponding to a Bernoulli random variable with generating function $G^B(s) = p s + (1 - p)$, the generating function for the reported offspring distribution becomes

6 $$\begin{aligned} G^D_n(s) = G_n \! \left( G^B(s) \right) = \left( 1 + \frac{p R}{k} (1-s) \right) ^{-k} = G_n(s ; p R, k). \end{aligned}$$

These formulas combined show that the reported offspring distribution from *M* people is $\text {NB}(p M R, M k)$.

### Base likelihood model

We derive our likelihood model by calculating the probability to observe a given number of cases on a specific day given the previous days’ case counts. Define the variable $z(d_1, d_2)$ as the number people reported on day $d_2$ whose infector was reported on day $d_1$. Using the above results we have

7 $$\begin{aligned} z(d_1, d_2) \sim \text {NB}(p(d_2 - d_1) \, c_1 \, R, c_1 \, k), \end{aligned}$$

where $c_1$ is the number of cases on day $d_1$, and $p(\Delta \tau )$ is the distribution of the time between reporting.

The number of infections $c_2$ on day $d_2$ is given by

8 $$\begin{aligned} c_2 = \sum _{d_1} z(d_1, d_2). \end{aligned}$$

In other words: the number of infections reported on day $d_2$ is the sum of those cases from the surrounding days ($\{d_1 \}$) that are reported on day $d_2$. We make the assumption that these are independent, although this is not strictly true. In this case $c_2$ will be distributed as

9 $$\begin{aligned} c_2 \sim {\mathop {\circledast }\limits _{d_1}} \text {NB}(p \, (d_2 - d_1) \, c_1 \, R, c_1 \, k), \end{aligned}$$

where $\circledast$ denotes convolution.

The total log likelihood is then found by summing over all regions and days:

10 $$\begin{aligned} \log {\mathcal {L}} = \sum _\text {regions} \sum _{i} \, \log \! \left( {\mathop {\circledast }\limits _{j}} \text {NB}(c_i \, ; \, p \, (d_i - d_j) \, c_j \, R, \, c_j \, k) \right) , \end{aligned}$$

where $c_i$ is the number of cases on day $d_i$. We use PyTorch to evaluate this expression and its gradients on an Nvidia Geforce RTX 2080 Ti GPU. More details are given in the SI.

## Supplementary information

Supplementary Information.
