# Supplementary material for: Superspreading quantified from bursty epidemic trajectories
Source: Sci Rep. 2021 Dec 16;11:24124. doi: 10.1038/s41598-021-03126-w (PMC8677763; doi:10.1038/s41598-021-03126-w)
Supplement: Supplementary file 1 — Supplementary Information. [file 41598_2021_3126_MOESM1_ESM.pdf]

# SI: Superspreading Quantified from Bursty Epidemic Trajectories

Julius B. Kirkegaard and Kim Sneppen
*Niels Bohr Institute, University of Copenhagen, 2100 Copenhagen, Denmark*
(Dated: October 7, 2021)

## CORRECTION FOR NUMBER OF TESTS

To correct for the number of tests in the distribution of

$$\Delta\tau = d_2 - d_1 = \tau_2 + \iota_2 - \tau_1, \tag{1}$$

we rescale $p(\tau_d)$ by the number of tests. Denote by $T(d)$ the number of tests on day $d$, then we redefine

$$p(\tau_d, d) = \frac{p(\tau_d)\, T(d)}{\sum_\delta p(\tau_d + \delta)\, T(d+\delta)}. \tag{2}$$

This allows us to calculate an updated version of $p(\Delta\tau)$ which now must be written as

$$p(d_1, d_2) \tag{3}$$

as it explicitly depends on the days.

To verify that this is the right correction to make, we consider, as discussed in the main text, the correlation between regions of the deviations of daily cases from a running mean. In the main text it is shown that $c_i(d) - \langle c_i(d)\rangle$ has a larger correlation coefficient than $c_i(D)/T_i(d) - \langle c_i(d)/T_i(d)\rangle$. One would generally not expect the number of reported cases to scale direct in proportion to the number of tests, however. Thus we also considered the correlation of $c_i(d)/T_i(d)^\gamma - \langle c_i(d)/T_i(d)^\gamma\rangle$ As shown in Fig. 1, we find the smallest correlation for exactly $\gamma \approx 1$ when we compare to a running mean on the scale of weeks.

## TOTAL LIKELIHOOD

Using $c_i(d)$ for the number of cases in region $i$ on day $d$, our total log likelihood is calculated as follows:

1. Assign parameters $R(t)$, $k$ and $c$.
2. For each region $i$:
   (a) Calculate the effective number of cases taking crossing into account: $c_i^{\text{eff}} = (1-c)\, c_i + c f_i C(d)$, where $f_i$ is the population fraction and $C(d)$ the total number of cases across all regions on day $d$.
   (b) For each day $d$:
      i. Calculate $p(d', d)$ using Eq. (2)
      ii. Use $p(d', d)$ of the main text to calculate $p(c_i^{\text{eff}}(d))$
3. Take the logarithm of $p(c_i^{\text{eff}}(d))$ and sum over all days and regions.

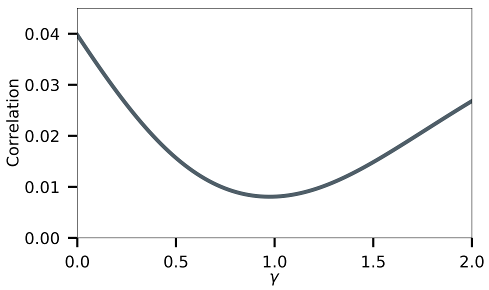


FIG. 1. Correlation of deviation in cases as a function of correction exponent $\gamma$. Best correction is found for $\gamma \approx 1$.

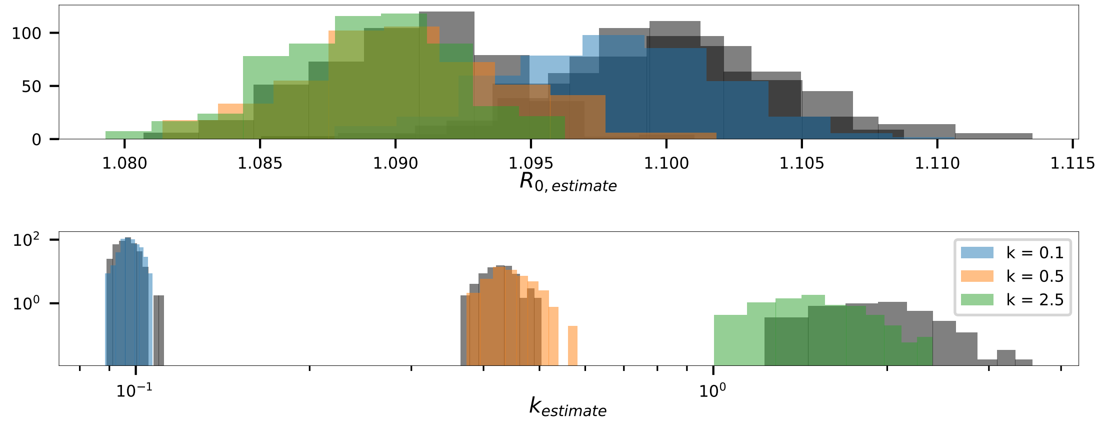


FIG. 2. Example of validation of model. MCMC on simulated data of 250 regions over 100 days with $R_0 = 1.1$ and $k$ as specified in legend. Simulations and MCMC were run with $p(\tau_i) \sim \Gamma(5.0, 10)$ and $p(\tau_d) \sim \Gamma(4.5, 5.0)$ and crossing parameter $c = 0.05$. Plot shows runs for two simulations for each $k$.

## CHECKS & CAVEATS

To check our method we compared it to a simulation. In the simplest case, this simulation was a run as a $\tau$-leaping stochastic simulation taking steps of one day:

1. Assign $R(t)$, $k$, $p(\tau_i)$ and $p(\tau_d)$.
2. Initialise a list of current infectious people and their infectivity $\boldsymbol{\nu} = [\nu_1]$ and day on which they got infected $\boldsymbol{d} = [d_1]$
3. For each day $d$
   (a) Calculate number of people infected on this day as $n_d = \sum_i X_i$, where $X_i$ is the number of people that person $i$ infects on day $d$, sampled from $\text{Pois}(\nu_i \cdot p(\tau_i = (d - d_i)))$.
   (b) For each newly infected people, update $\boldsymbol{\nu}$ and $\boldsymbol{d}$ with new infectivities $\nu_j$ sampled from $\Gamma(R(d), k)$.
4. For each person, sample their day of reporting of infection from $p(\tau_d)$.

We ran these simulations using 100 regions over 250 days, matching the data used in the main text. Using our likelihood model, we were consistently able to reproduce $R$ and $k$ to within a few percent of the simulations despite the assumptions used to derive our model. We also tested the effect of including imported cases using a crossing parameter. See Fig. 2 for an example.

Finally, we subsampled our simulations using a binomial distribution to simulate the effects of under-reporting. The result is shown in Fig. 3.

To test the dependency of our results on specific municipalities we ran our model with only half of all municipalities sampled randomly. All test such test runs returned similar results. We further tested explicitely excluding the capital region (Copenhagen, Frederiksberg, Gentofte, Dragør, Vallensbæk, Gladsaxe, Herlev, Hvidovre, Rødovre, Brøndby, Glostrup, Høje, Taastrup Tarnby, Ishøj and Albertslund) which again did not make a significant difference.

Restricting our model to subsets of the full time interval naturally results in variation. Solving for the maximum likelihood of $R$ and $k$ we found variations not just for $R$, but also the dispersion parameter $k$, which (for fixed distributions of $\Delta\tau$) varied between $0.08 - 0.16$.

In closing we note that our analysis depends on the following assumptions:

- We assume the value for $R$, $k$, $c$ and distributions of $\Delta\tau$ equal for all municipalities. It will most likely be the case that these vary slightly from region to region.
- The number of imported cases in each municipality is defined by a crossing parameter that couples all municipalities. Our model thus does take into account the geography. A simple extension of our model would make the cross parameter $c$ depend on the distance between municipalities. This will likely change the value of $c$, but only slightly affect $R$ and $k$.

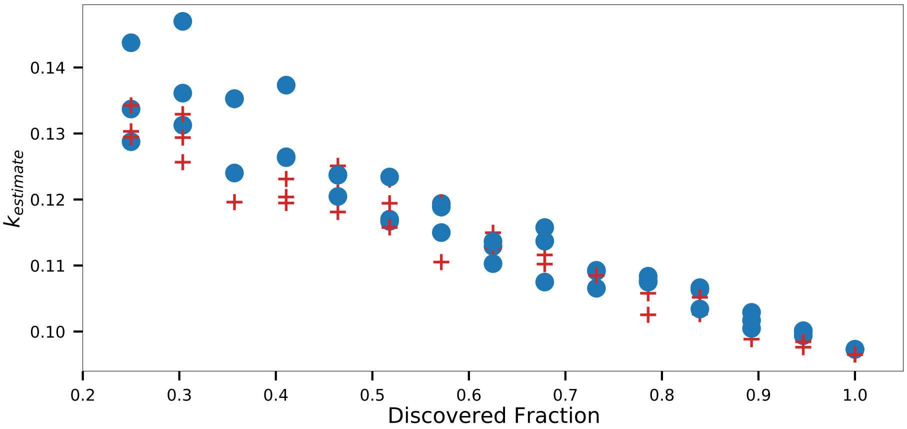


FIG. 3. The maximum likelihood $k$ estimated for simulation that have subsequently been subsampled by a binomial distribution. Each point is one estimate and circles and crosses represent two different simulations. We ran the analysis three times for each simulation of reported fraction to visualise the noise of the binomial distribution.

- We assume that $R(t)$ varies slowly. Interventions can in principle make sudden changes to $R(t)$.
- We assumed independence of the random variables to calculate the total likelihood. As mentioned in the main text, this will cause our distribution of $\Delta\tau$ to be too wide. We tested the effect of this by considering narrower distributions.
- We assume that municipalities are uncorrelated except for their testing frequency. After correcting for testing we, nonetheless, measure a correlation coefficient of $\sim 0.01$ as shown in Fig. 1.

## CODE

An implementation of our model is available at `https://github.com/juliusbierk/superspreadingdispersion` along with a simple example of how to use the model on data. A GPU is needed for reasonable speed of execution.
